# Genome Sequence of *Arthrobacter globiformis* B-2979 Phage Raphaella

**DOI:** 10.17912/micropub.biology.001464

**Published:** 2025-03-28

**Authors:** Hannah Alapati, Adam Parks, Tyler Hildebrand, Joshua Leazer, Kateryn Rodriguez, John Patton

**Affiliations:** 1 Natural and Applied Sciences, Evangel University, Springfield, Missouri, United States

## Abstract

Bacteriophage Raphaella was isolated from a soil sample collected in Springfield, MO using *Arthrobacter globiformis* B2979-SEA *.* Raphaella has a genome of 51692 base pairs with a GC content of 62.6%, 96 putative protein encoding genes and one tRNA. It has been placed in the AY cluster of Actinobacteriophages based on gene content similarity.

**Figure 1. Plaque and virion morphology:**
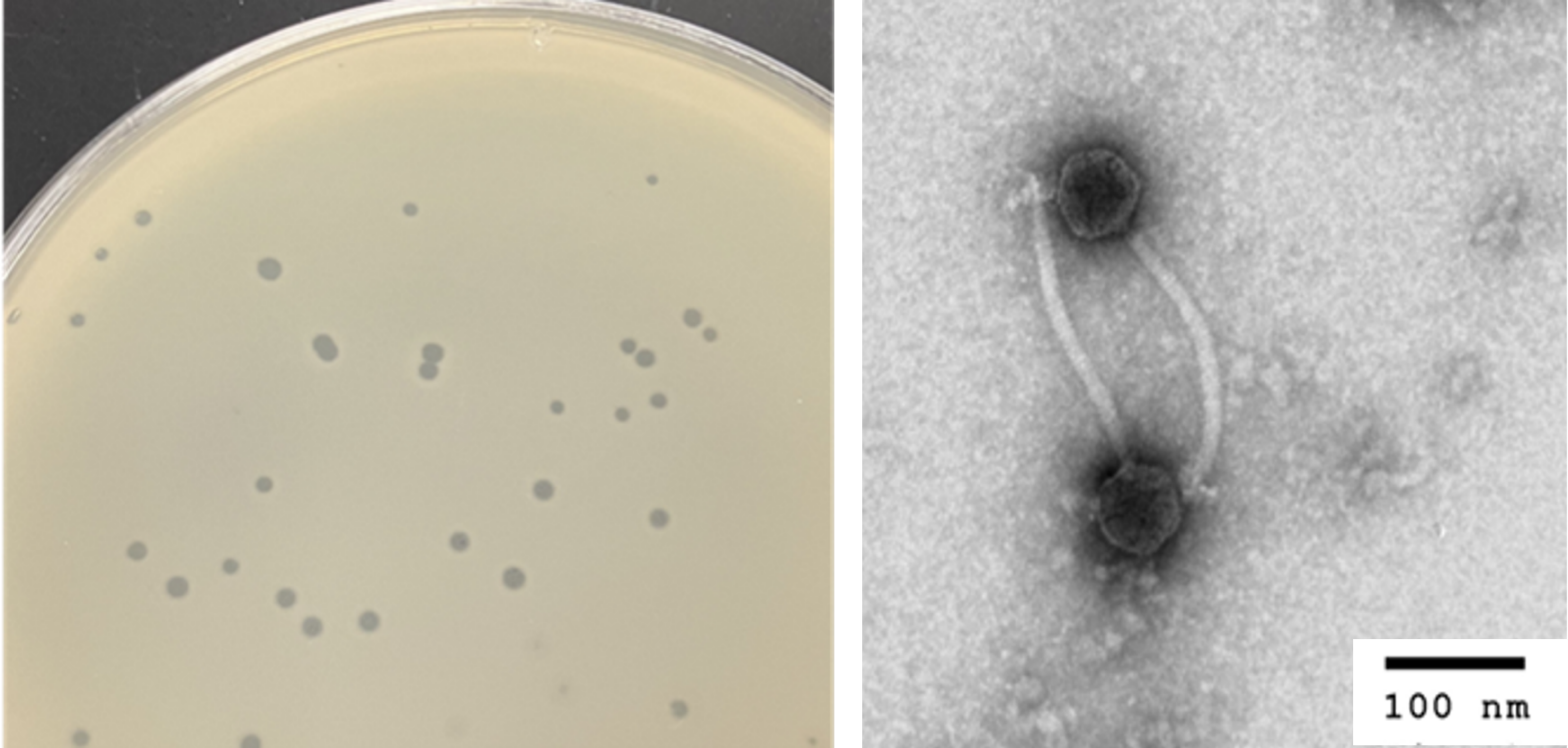
Left: Raphaella generates clear plaques with well-defined borders. Right: Raphaella particles have siphovirus morphology, with a polyhedral capsid and long, flexible tail.

## Description

As antibiotic-resistant bacterial infections continue to rise, bacteriophages are being developed as an alternative therapeutic (Sulakvelidze et al., 2001, Strathdee and Patterson, 2019). In support of this development, the discovery and genetic characterization of novel phages is invaluable. Here, we report on the novel phage, Raphaella, which was isolated in September 2023, from a soil sample collected at Valley Water Mill Park in Springfield, MO (GPS: 37.26409 N, 93.24684 W). The soil sample was wet and contained small roots. This sample was suspended in peptone-yeast calcium (PYCa) liquid media, and the suspension was then centrifuged (2,000 x g, 10 min). The supernatant was filtered (0.2 micron pore size) before the filtrate was inoculated with *Arthrobacter globiformis* B2979-SEA. Following 3 days of incubation at 30˚C with shaking, an aliquot of the resulting culture was filtered. The filtrate plated in top agar with *A. globiformis* , giving rise to plaques of Raphaella after incubation of plates at 30˚C for 3 days. Raphaella was purified through three rounds of plating (Zorawik et al., 2024). Raphaella forms clear plaque with a diameter of 0.8 +/- 0.24 mm(n=5) (Zorawik et al., 2024) (Figure 1). A lysate was prepared and used for imaging virion particles by transmission electron microscopy using negative staining (1% uranyl acetate), revealing a capsid 59.2 +/- 1.9 nm (n=5) wide and the length of the tail was 210.7 +/- 15.8 nm (n=5) (Figure 1).

DNA of Raphaella was extracted from the lysate utilizing the Promega Wizard DNA clean-up kit, then sequenced on an Illumina MiSeq with v3 reagents after preparation with a NEB Ultra II Library Kit, which yielded 618710, 150 bp reads which constituted approximately 1701-fold coverage. These reads were assembled using Newbler v2.9 into a 51692 bp genome with 62.6% GC content, with 3' single-stranded genome termini determined using Consed v29 (Russell, 2018, Gordon and Green, 2013).

Raphaella's genome was automatically annotated using DNA Master v5.23.6 (Pope and Jacobs-Sera, 2018), embedded with Genemark v2.5 (Besemer and Borodovsky, 2005) and Glimmer v3.02 (Delcher et al., 2007). Translational starts were determined manually using the coding potential predicted in GeneMark (Besemer and Borodovsky, 2005) and then refined by comparison with similar genes using Starterator v578 (http://phages.wustl.edu/starterator/) and Phamerator v 578 (Cresawn et al., 2011). Putative functions were assigned to the genes using PECAAN (discover.kbrinsgd.org) and in the embedded BLASTp (Altschul et al., 1990) searches against the NCBI protein database and the actinobacteriophage database (Russell and Hatfull, 2016) as well as HHPred using PDB_mmCIF70, SCOPe70, Pfam-A, NCBI_Concerved_Domains (CD) databases (Söding et al., 2005). Utilizing ARAGORN v1.2.41 (Laslett and Canback, 2004) and tRNA scan v2.0 (Lowe and Eddy, 1997), a single tRNA which coded for the amino acid glycine was identified. Nine potential membrane proteins were found using DeepTMHMM v1.0 (Jeppe et al., 2022). All software were used with default setting. The annotation process revealed a total of 96 genes, 39 of which could be assigned putative functions. Based on gene content similarity of over 35% to phages in the Actinobacteriophage database, phagesDB, Raphaella was assigned to the AY cluster (Russell and Hatfull, 2016)

As with a majority of cluster AY phages, Raphaella encodes two tyrosine integrases. This, coupled with experimental evidence of lysogen formation by other cluster AY phages, suggests that Raphaella too is likely to establish lysogeny. We note, however, that no immunity repressor function could be predicted in Raphaella.

Nucleotide Sequence Accession numbers:

Raphaella is available at GenBank with Accession Number PP987873 and Sequence Read Archive Number SRX26311147 .
